# Evaluation of Corneal Topography and Biomechanical Parameters after Use of Systemic Isotretinoin in Acne Vulgaris

**DOI:** 10.1155/2014/701361

**Published:** 2014-11-30

**Authors:** Yusuf Yildirim, Onur Olcucu, Alper Agca, Cengiz Alagoz, Ali Demircan, Abdurrahman Basci, Ahmet Demirok, Zekayi Kutlubay

**Affiliations:** ^1^Department of Ophthalmology, Beyoglu Eye Research and Education Hospital, Bereketzade Cami Sokak No. 2, Beyoğlu, 34420 Istanbul, Turkey; ^2^Department of Ophthalmology, Göztepe Education and Research Hospital, Istanbul Medeniyet University, Dr. Erkin Caddesi, Kadıkoy, 34732 Istanbul, Turkey; ^3^Department of Dermatology, Cerrahpaşa Faculty of Medicine, Istanbul University, Kocamustafapaşa Caddesi No. 53, Cerrahpaşa, Fatih, 34098 Istanbul, Turkey

## Abstract

*Purpose*. We report the effect of isotretinoin on corneal topography, corneal thickness, and biomechanical parameters in patients with acne vulgaris.* Method*. Fifty-four eyes of 54 patients who received oral isotretinoin for treatment of acne vulgaris were evaluated. All patients underwent a corneal topographical evaluation with a Scheimpflug camera combined with Placido-disk (Sirius), ultrasonic pachymetry measurements, and corneal biomechanical evaluation with an ocular response analyzer at baseline, in the 1st, 3rd, and 6th months of treatment, and 6 months after isotretinoin discontinuation.* Results*. The thinnest corneal thickness measured with Sirius differed significantly in the 1st, 3rd, and 6th months compared with the baseline measurement; there was no significant change in ultrasonic central corneal thickness measurements and biomechanical parameters (corneal hysteresis and corneal resistance factor) throughout the study. Average simulated keratometry and surface asymmetry index increased significantly only in the first month of treatment according to the baseline. All changes disappeared 6 months after the end of treatment.* Conclusion*. Basal tear secretion and corneal morphologic properties were significantly influenced during the systemic isotretinoin treatment and the changes were reversible after discontinuation. No statistical important biomechanical differences were found to be induced by isotretinoin.

## 1. Introduction

Oral isotretinoin has been used for almost three decades for the treatment of severe recalcitrant nodular acne and various other skin disorders. Isotretinoin affects all of the major aspects of acne. The inhibition of the cell differentiation of sebaceous glands results in a significant reduction in sebum production, which influences comedogenesis and lowers surface and ductal* P. acnes*. A significant reduction in the* P. acnes* population and the modification of monocyte chemotaxis precipitates the reduction in acne inflammation [1].

The most common adverse ocular reactions associated with isotretinoin are based on its mode of action in the treatment. Similar to skin sebaceous glands, isotretinoin also affects the meibomian glands in the eyes and causes significant meibomian gland atrophy. An evaporative form of ocular sicca, blepharoconjunctivitis, meibomitis, photodermatitis of the eyelids, corneal opacities, contact lens intolerance, blurred vision, decreased dark adaptation, myopia, keratitis, and premacular hemorrhage are additional ocular adverse effects observed during systemic isotretinoin treatment [2–4].

Isotretinoin therapy may result in dry eye and the effects of oral isotretinoin on ocular surface and tear film are well defined [5, 6]. We have not focused on ocular surface and dry eye in this study. However, tear break-up time (TBUT), Schirmer 1 test (S1T) without anesthesia, and an anesthetized Schirmer test (AST) before, during, and after isotretinoin therapy were evaluated to report potential complications. To the best of our knowledge, the effects of oral isotretinoin on corneal biomechanics have not been studied before. This study was carried out to investigate corneal biomechanical and topography changes in patients treated with oral isotretinoin.

## 2. Materials and Methods

This prospective and observational study was performed at the Ophthalmology Department of the Beyoglu Eye Education and Research Hospital and the Department of Dermatology of Cerrahpasa Faculty of Medicine, Istanbul University. Fifty-eight eyes of 58 patients with acne vulgaris treated with oral isotretinoin (0.8 mg/kg daily) were enrolled in this study. The cumulative dose of isotretinoin was lower than 120 mg/kg in all patients. The right eye of each participant was used for analyses. The study was performed over 20 months (December 2011 through July 2013).

The main inclusion criteria for enrollment were an age above 18 years and a diagnosis of acne vulgaris with planned oral isotretinoin treatment. Patients with a history of previous ocular surgery or eye trauma, contact lens use, ocular inflammatory disease, any systemic disease except acne vulgaris, or a history of using ocular or systemic medications (except for isotretinoin) within the previous 3 months were excluded from study. Furthermore, women who were pregnant, planning a pregnancy, or lactating were excluded. Hemogram, liver enzymes, triglycerides, total cholesterol, and lipoprotein levels were followed up on throughout the study for detecting any possible systemic adverse effects.

The study followed the tenets of the Declaration of Helsinki and was approved by the local ethics committee. All participants received oral and written information about the study, and each participant provided a written informed consent form.

Three independent experienced technicians performed each of the techniques and all were blinded to the results of the previous tests. Scheimpflug camera combined Placido-disk corneal topography (Sirius, Costruzione Strumenti Oftalmici, Scandicci, Italy) measurements were always performed first. Next, another technician assessed the corneal biomechanical properties by means of an ocular response analyzer (ORA) (Reichert Ophthalmic Instruments, Buffalo, NY, USA). After 5 minutes, the study participants underwent ultrasonic central corneal thickness (CCT) measurements with another technician using a SP100 Handy pachymeter (Tomey, Nagoya, Japan). A drop of 0.5% proparacaine hydrochloride (Alcaine, Alcon Couvreur, Puurs, Belgium) was instilled to the eye before the ultrasonic pachymetry readings were taken. Before each measurement, all of the instruments were calibrated with the use of tests recommended by the manufacturer. The mean of three repeated measurements was calculated.

One day later, for the measurements discussed above, a complete ophthalmologic examination was performed by the same ophthalmologist. This ophthalmologic examination was repeated, during the 1st, 3rd, and 6th months of the treatment plan and 6 months after the cessation of treatment for all patients.

Each clinical examination included best-corrected visual acuity using a Snellen chart, intraocular pressure measurement by a Goldmann applanation tonometer, biomicroscopy of the anterior segment, and dilated fundus examination. The S1T, ASA scores, and TBUT were also recorded during the examination.

A S1T was performed for the evaluation of the basal and reflex tear secretion. Excess wetness on the eyelid margin was wiped with a cotton-tipped applicator. A Schirmer test strip (Clement Clarke, Edinburg, United Kingdom) was placed at the inferotemporal fornix in order to avoid touching the cornea and the amount of wetting from the folded end was measured in millimeters and recorded as the S1T score of the participants after 5 minutes.

To measure basal tear secretion, an AST was carried out by adding a drop of 0.5% proparacaine hydrochloride twice into the inferior fornix of the right eye at 5-minute intervals. After drying the excess wetness a Schirmer test strip was placed and the AST score was recorded in the same way as the S1T result.

TBUT was determined by applying a fluorescent strip moistened with distilled water (Haag-Streit AG, Köniz, Switzerland) to the lower conjunctival fornix and thereafter observing the tear film using the cobalt blue filter of the slit-lamp while the patient avoided blinking until the first dry spot developed. This procedure was repeated 3 times and the average time between the last blink and appearance of the first dry spot was recorded as break-up time value.

All statistical analyses were performed using the Statistical Package for the Social Sciences (SPSS) version 16 (SPSS Inc., Chicago, IL, USA). The normality of the data was confirmed using a Kolmogorov-Smirnov test (*P* > 0.05). A two-paired* t*-test was used to compare the study measurements at the baseline, three follow-up visits, and 6 months after the end of the treatment. Differences with a value of *P* < 0.05 were considered to be statistically significant.

## 3. Results

In this study, 58 eyes of 58 patients were enrolled. Fifty-four of the 58 patients completed the study; 4 patients were excluded due to abnormal corneal findings at their baseline visits (2 patients had corneal opacities, 1 patient had corneal dystrophy, and 1 patient had undergone previous LASIK surgery).

The mean ± standard deviation of the ages of the 34 women and 20 men was 23.9 ± 4.1 years, with a range of 18–37 years.

### 3.1. Tear Film Break-Up Time and Schirmer Tests

AST scores and TBUT values decreased significantly in the 1st, 3rd, and 6th months of treatment, respectively, compared with baseline values. There were no significant changes in the AST scores and TBUT values between the baseline and 6 months after the cessation of treatment. We found no statistically significant difference in ST1 scores at the baseline, during the treatment, and after the completion of treatment. The AST, ST1 scores, and TBUT values are listed in Table 1.

### 3.2. Corneal Topographical Findings

The Sirius findings of patients at baseline, during treatment, and 6 months after treatment are listed in Table 2. The thinnest pachymetries were statistically significantly decreased between the baseline and 1st, 3rd, and 6th months of treatment. The average simulated keratometry values (SimK) and surface asymmetry index (SAI) were significantly different at the first month of treatment but did not differ after 3 and 6 months compared with the baseline value. All statistically significant changes were reversible and no difference was found between the baseline value and the value recorded 6 months after the discontinuation of isotretinoin.

### 3.3. Biomechanical Parameters

Corneal hysteresis (CH), corneal resistance factor (CRF), and CCT measured with ultrasonic pachymetry were not significantly different throughout the study (*P* > 0.05). The ultrasonic CCT measurements and corneal biomechanical parameters of patients assessed with ORA before, during, and after treatment are listed in Table 3.

## 4. Discussion

Oral isotretinoin has been widely used in medical treatments associated with patients with recalcitrant nodulocystic acne since the approval of the medicine by the US Food and Drug Administration in 1982. Isotretinoin results in long term remission of acne, with the inhibition of many contributing factors in the genesis of acne and remains the most clinically effective acne treatment of the past three decades. It strongly affects the sebaceous glands in the skin and leads to an 80% reduction in sebum excretion and comedogenesis. It also inhibits the chemotaxis and colonization of* P. acnes* with anti-inflammatory properties [1].

Isotretinoin can adversely affect the function of the meibomian glands due to similarities between the meibomian glands and sebaceous glands in the skin. Lambert and Smith reported a decrease in the density of goblet cells and the size of the acini and the lipid content of the meibomian glands in rabbit models [7]. Mathers et al. confirmed this result by performing meibography in humans [8]. The lipid layer is composed of the meibomian lipids and its thickness and likely its composition influence the rate of evaporation [9]. Karalezli et al. reported a statistically significant decrease in AST scores for 50 subjects treated with 0.8 mg/kg of oral isotretinoin [5]. Cumurcu et al. found a significant decrease in TBUT values for both high doses (>0.5 mg/kg per day) and low doses (<0.5 mg/kg per day) of systemic isotretinoin administrated to patient groups [6]. In our study, the statistically significant decrease in TBUT and AST scores, which indicates basal tear secretion, was consistent with these previous studies.

In cataract surgery, three parameters are crucial in order to predict correctly the patient's refractive outcome: the axial length of the eye, the keratometry readings of the cornea, and the predicted position of the intraocular lens after surgery [10]. SimK and SAI are essential indices of Sirius for corneal topography. SimK provides the power and axis of the steepest and flattest meridian similar to values provided by the keratometer. It is calculated from rings 7 to 9 corresponding to the position on the cornea at which the keratometry measurements were obtained. SAI is a centrally weighted summation of differences in corneal power between corresponding points 180° apart on 128 equally spaced meridians. We found a statistically significant increase for only average SimK values and SAIs between the baseline and the first month of isotretinoin treatment with the use of Sirius. Cumurcu et al. recorded significant changes in mean keratometry values between baseline and after the 3rd and 6th months of therapy by means of Pentacam [11]. The increase in keratometry readings of cornea should be considered when calculating the intraocular lens power for patients at early stages of isotretinoin therapy.

These findings highlight changes in corneal topography in patients who use isotretinoin, which are important considering that these patients are often young and potential candidates for refractive surgery. The precise assessment of these parameters is required for the appropriate management of patients, and any factors that affect corneal topography should be known by clinicians. We found a significant reduction in the thinnest corneal thickness measured with Sirius between the baseline and the 1st, 3rd, and 6th months of isotretinoin treatment. Similarly, Cumurcu et al. found a significant difference between pretreatment values and the 3rd and 6th month values for CCT, as measured with a Pentacam rotating Scheimpflug, in patients treated with isotretinoin [11]. A value of residual stromal bed thickness below 250 *μ*m is considered to be a major risk factor for the development of iatrogenic keratectasia in corneal refractive surgery [12]. Since the residual stromal bed thickness is dependent on preoperative CCT, it may be reasonable to suggest that isotretinoin usage could result in an erroneous calculation of the residual stromal thickness of patients at the preoperative evaluation. The pachymetry measurements and corneal topographic evaluations were only performed at 6th months after isotretinoin discontinuation in our study. We were therefore unable to investigate these changes at early times after isotretinoin discontinuation (1st and 3rd months) which may be a drawback of our study.

S1T evaluates the volume of tears produced after irritation of the ocular surface via filter paper strips. Mathers et al. reported on 11 patients treated with systemic isotretinoin and found no statistically significant difference in S1T scores during the therapy [8]. Similarly, Bozkurt et al. evaluated 40 patients treated with systemic isotretinoin and found no statistically significant difference in S1T values [13]. We detected a mild reduction in S1T scores, but the decrement was not statistically significant.

The labile tear lipid layer induces increasing evaporation and tears osmolarity [14]. Liu and Pflugfelder reported that the chronic state of desiccation and immune activation in dry eyes may contribute to central and midperipheral corneal thinning, thereby decreasing CCT [15]. Niimi et al. revealed that increased tear osmolarity causes corneal deswelling and decreased central thickness [16]. This mechanism may be a contributing factor to the decreased pachymetry measurements recording during the isotretinoin treatment in the current study. Although the ultrasonic CCT measurements were slightly lowered, the differences were not statistically significant. Mukhopadhyay et al. evaluated corneal thickness using scanning-slit (Orbscan IIz) and Scheimpflug devices (Pentacam) before and after the administration of 0.5% proparacaine and concluded that ultrasound pachymetry, combined with 0.5% proparacaine, caused a small (<10 *μ*m) but significant level of corneal swelling on average [17]. The difference in CCT measurements between the two devices was likely caused by the use of the topical anesthetic agent in the current study. Consistent with our results, Jorge et al. reported statistically significant differences in the CCT values measured with the Sirius and ultrasound pachymetry [18].

Despite the importance of the ocular surface disease index (OSDI) and the staging of the ocular surface by staining by vital dyes such as rose bengal, fluorescein, or lissamine green for diagnosis, staging, and treatment follow-up of the diseases of the ocular surface, a major limitation of this study is the lack of OSDI scores and the evaluation of ocular surface using vital dyes [19, 20]. This limitation primarily arose because the focus of this study was on changes in corneal topography and the biomechanics of patients treated for acne vulgaris. A large number of studies about the effects of oral isotretinoin on ocular surface and tear films have been published in recent years [5, 6]. S1T, AST, and TBUT scores are only followed up for early detection of possible severe dry eye related to isotretinoin administration and to determine the artificial tear need of participants.

The precorneal tear film plays an important role in corneal stability and integrity. Fraunfelder reported corneal problems, such as keratitis, corneal opacity, corneal ulceration, and keratoconus, as the second most prevalent adverse effect among patients during isotretinoin treatment [21]. Firat and Doganay conducted a study in patients with dry eye and concluded that CH and CRF are not influenced by dry eye [22]. We would like to investigate whether the changes in precorneal tear film properties induced by isotretinoin have an effect on the corneal biomechanics measured with ORA. There were no statistically significant changes in corneal hysteresis or corneal resistance factors in our study. All participants were monitored for any ocular or systemic adverse effects associated with isotretinoin during the study. Fraunfelder reported blepharoconjunctivitis or meibomitis in 37% of patients, blurred vision in 17% of patients, and corneal opacities in 5% of patients [21]. The side effects we encountered were blepharoconjunctivitis and meibomitis in 19 of 54 patients (35%) in our study. These findings are consistent with previous studies. Some transient visual deteriorations were stated in daily activities from the beginning of treatment by 9 patients (16%), but we could not determine any decrease in visual acuity based on clinical visits. We can speculate that the changes in visual acuity in daily activities as stated by the patients may be attributable to tear film instability.

In conclusion, isotretinoin treatment causes statistically significant changes in the tear properties, secretion, and ocular surface, but it does not have any effect on corneal biomechanics. Due to its effects on corneal properties, clinicians should avoid performing any surgeries that require an accurate knowledge of corneal parameters, such as keratometry readings and corneal thickness, at least 6 months after the cessation of isotretinoin use. The ocular adverse reactions associated with isotretinoin are frequent but generally not serious and are reversible after the end of treatment.

## Figures and Tables

**Table 1:** Changes of Schirmer 1 test, anesthetized Schirmer test, and tear break-up time of the patients.

	Baseline	1 month	3 months	6 months	Follow-up
S1T, mm/5 min					
Mean ± SD	28.80 ± 2.40	27.60 ± 2.35	26.88 ± 2.08	26.32 ± 1.96	28.78 ± 2.38
*P* ^a^		0.132	0.116	0.094	0.854
AST, mm/5 min					
Mean ± SD	18.74 ± 3.42	16.96 ± 3.30	15.06 ± 3.13	14.92 ± 3.22	18.72 ± 3.40
*P* ^a^		0.044^∤^	0.021^∤^	0.008^∤^	0.564
TBUT, sn					
Mean ± SD	14.84 ± 2.11	10.44 ± 2.82	9.86 ± 2.92	9.42 ± 3.11	14.76 ± 2.26
*P* ^a^		0.012^∤^	0.008^∤^	0.006^∤^	0.342

*P* ^a^, *P* value according to paired *t*-test compared with baseline.

S1T, Schirmer 1 test; AST, anesthetized Schirmer test; TBUT, tear break-up time; SD, standard deviation.

^∤^Statistically significant at the 0.05 level.

**Table 2:** Corneal topographical parameters measured with Sirius before and after treatment.

	Baseline	1 month	3 months	6 months	Follow-up
Average SimK, D					
Mean ± SD	43.68 ± 1.38	43.73 ± 1.35	43.62 ± 1.37	43.58 ± 1.38	43.66 ± 1.38
*P* ^a^		0.030^∤^	0.322	0.462	0.564
SAI					
Mean ± SD	0.39 ± 0.31	0.45 ± 0.33	0.44 ± 0.32	0.42 ± 0.33	0.40 ± 0.30
*P* ^a^		0.015^∤^	0.052	0.060	0.124
Thinnest CT, *μ*m					
Mean ± SD	539.51 ± 48.33	537.13 ± 46.77	535.66 ± 46.31	534.87 ± 46.23	539.46 ± 48.36
*P* ^a^		0.014^∤^	0.011^∤^	0.005^∤^	0.128

*P* ^a^, *P* value according to paired *t*-test compared with baseline.

SimK, simulated keratometry; SAI, surface asymmetry index; CT, corneal thickness; SD, standard deviation.

^∤^Statistically significant at 0.05 level.

**Table 3:** Biomechanical parameters measured with ocular response analyzer and central corneal thickness measured with ultrasonic pachymeter before and after treatment.

	Baseline	1 month	3 months	6 months	Follow-up
CH, mmHg					
Mean ± SD	11.67 ± 2.22	11.48 ± 2.13	11.50 ± 2.12	11.51 ± 2.13	11.58 ± 2.18
*P* ^a^		0.206	0.289	0.343	0.664
CRF, mmHg					
Mean ± SD	11.11 ± 2.33	11.05 ± 2.15	11.43 ± 2.14	10.97 ± 2.08	10.86 ± 2.04
*P* ^a^		0.691	0.644	0.535	0.489
CCT_usg_, *µ*m					
Mean ± SD	544.56 ± 46.14	542.18 ± 45.92	540.72 ± 45.94	539.89 ± 45.88	544.53 ± 46.08
*P* ^a^		0.547	0.322	0.208	0.782

*P* ^a^, *P* value according to paired *t*-test compared with baseline.

CH, corneal hysteresis; CRF, corneal resistance factor; CCT_usg_, central corneal thickness measured with ultrasonic pachymeter; SD, standard deviation.
